# Supplementary material for: Temperatures above 37°C increase virulence of a convergent Klebsiella pneumoniae sequence type 307 strain
Source: Front Cell Infect Microbiol. 2024 Jun 7;14:1411286. doi: 10.3389/fcimb.2024.1411286 (PMC11211929; doi:10.3389/fcimb.2024.1411286)
Supplement: Supplementary file 1 [file DataSheet_1.docx]

Supplementary Material

Temperatures above 37°C increase virulence of a convergent *Klebsiella pneumoniae* sequence type 307 strain

**Justus U. Müller^1^, Michael Schwabe^1^, Lena-Sophie Swiatek^1^, Stefan E. Heiden^1^, Rabea Schlüter^2^, Max Sittner^1^, Jürgen A. Bohnert^3^, Karsten Becker^3^, Evgeny A. Idelevich^3,4^, Sebastian Guenther^5^, Elias Eger^1†^, Katharina Schaufler^1,6†*^**

^1^Department of Epidemiology and Ecology of Antimicrobial Resistance, Helmholtz Institute for One Health, Helmholtz Center for Infection Research HZI, Greifswald, Germany

²Imaging Center of the Department of Biology, University of Greifswald, Greifswald, Germany

^3^Friedrich Loeffler-Institute of Medical Microbiology, University Medicine Greifswald, Greifswald, Germany

^4^Institute of Medical Microbiology, University Hospital Münster, Münster, Germany.

^5^Pharmaceutical Biology, Institute of Pharmacy, University of Greifswald, Greifswald, Germany

^6^University Medicine Greifswald, Greifswald, Germany

† These authors contributed equally to this work and share last authorship

*** Correspondence:**Corresponding Author
Katharina Schaufler, Department of Epidemiology and Ecology of Antimicrobial Resistance, Helmholtz Institute for One Health, Helmholtz Centre for Infection Research HZI, Greifswald, Germany, Phone: +49 3834-3916-200; E mail: katharina.schaufler@helmholtz-hioh.de

Table of Contents

[1.1 Supplementary Figures 3](#_Toc166592004)

[Supplementary Figure S1: Long term colonies on Spanangar plates 3](#_Toc166592005)

[Supplementary Figure S2: Principal component analysis (PCA) of the RNA sequencing output. 3](#_Toc166592006)

[Supplementary Figure S3: The *Galleria mellonella* *in vivo* infection model with mock-infection (PBS) 4](#_Toc166592007)

[Supplementary Figure S4: Correlation analysis between different temperatures to reveal the transcriptomic shift of 28 °C, 40 °C and 42 °C in comparison to 37 °C 4](#_Toc166592008)

[Supplementary Figure S5: RNA sequencing shows varying numbers of differentially expressed genes 5](#_Toc166592009)

[Supplementary Figure S6: Different temperatures affect mucoviscosity and overall virulence of the convergent *K. pneumoniae* ST307 strain PBIO1953. 5](#_Toc166592010)

[Supplementary Figure S7: 6](#_Toc166592011)

[2 Supplementary Tables 7](#_Toc166592012)

[Supplementary Table 1. Primer Sequences for plasmid copy number analyses of plasmid 1- 3, and chromosome. 7](#_Toc166592013)

[Supplementary Table 2. qPCR reaction setup per well. 7](#_Toc166592014)

[Supplementary Table 3. qPCR thermocycler protocol for the Biorad DFX Optus 96-well, qPCR Luna Mastermix. 8](#_Toc166592015)

[3 Supplementary methods 8](#_Toc166592016)

[Biofilm staining 8](#_Toc166592017)

[4 Supplementary literature 8](#_Toc166592018)

## Supplementary Figures


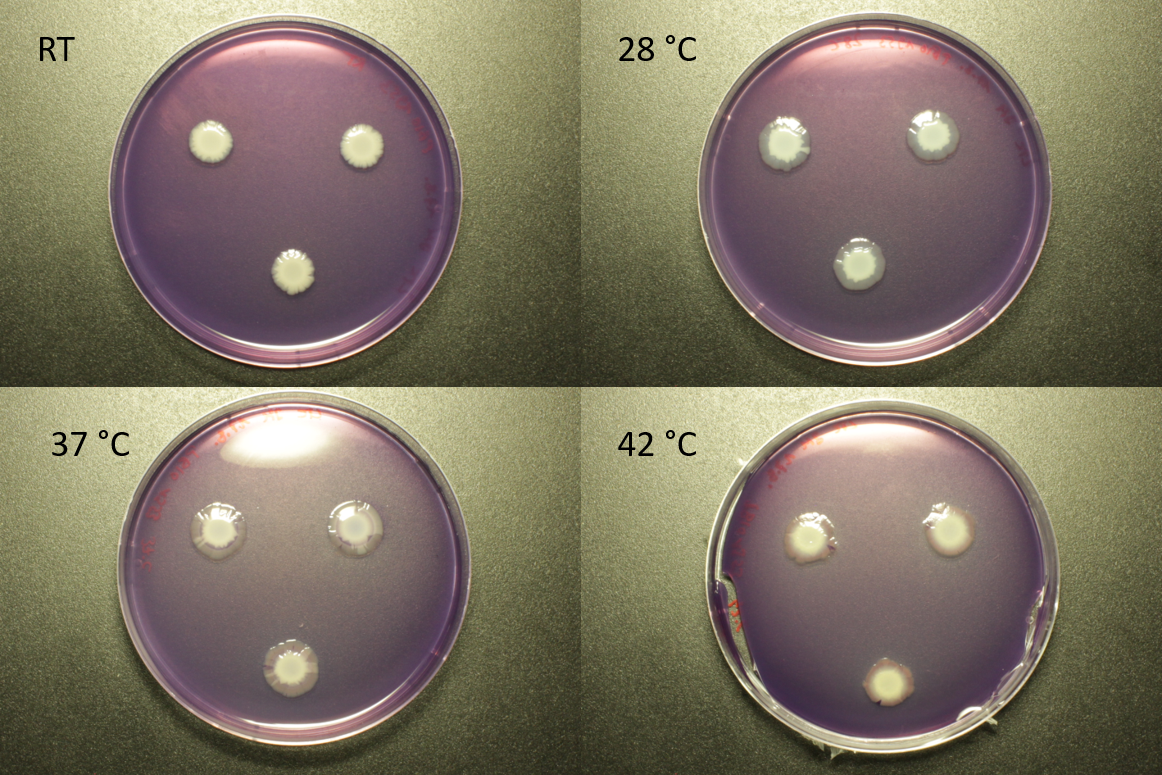


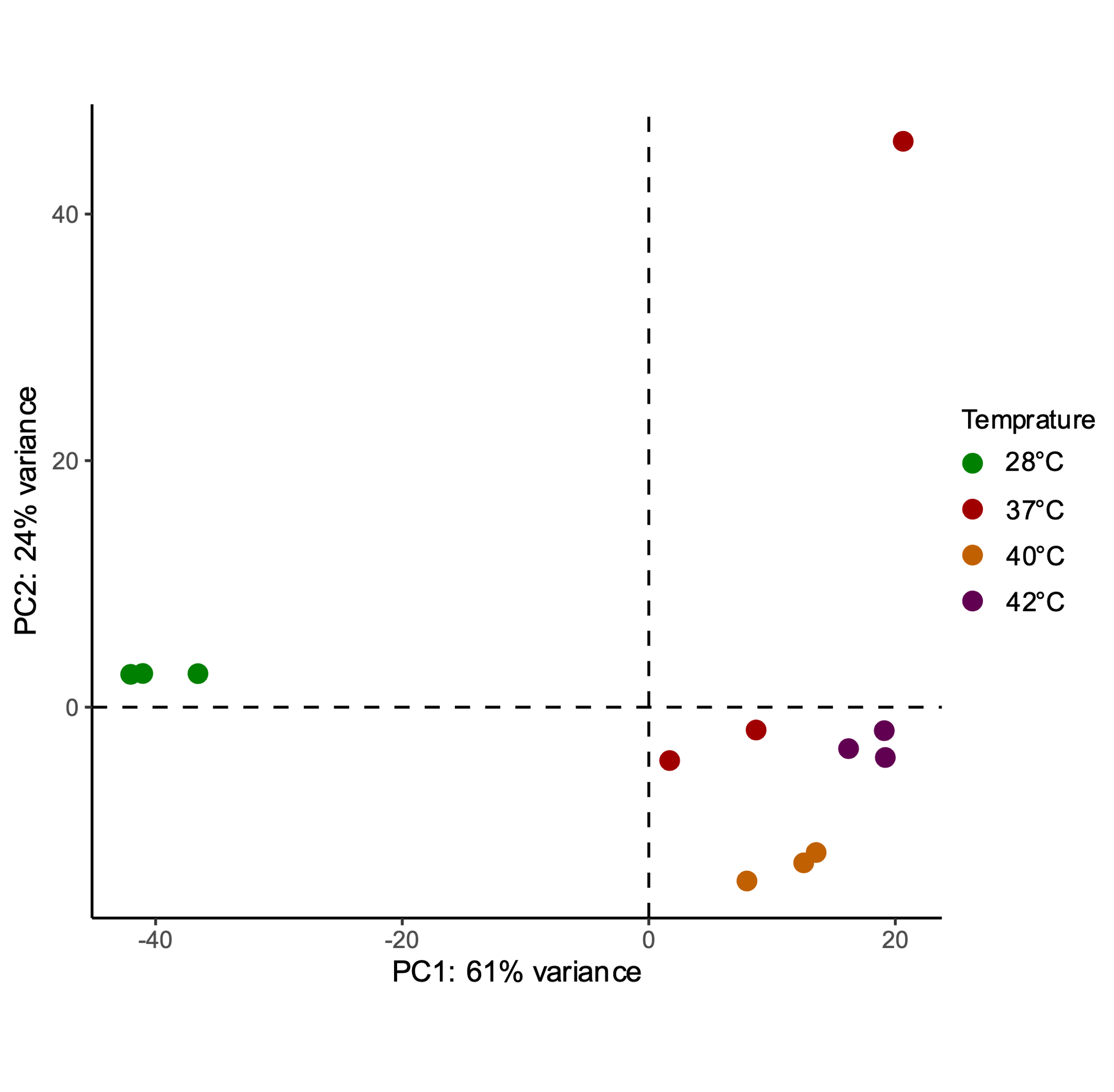
Supplementary Figure S1: Long term colonies on Spanangar plates after 5 days at RT, 28 °C, 37 °C and 42 °C. Colorless / white indicates no curli nor cellulose production suggesting a negative biofilm and no multicellular way of life independent of the temperature.

Supplementary Figure S2: Principal component analysis (PCA) of the RNA sequencing output. The PCA plot for the variance-stabilized transformation of the DESeq2 object was calculated on the basis of 1,000 top genes. We removed one replicate of the 37 °C (red) DESeq2 analysis because visualization revealed a shift on PC1 and PC2. Replicates belonging to one temperature group are indicated in the same color.


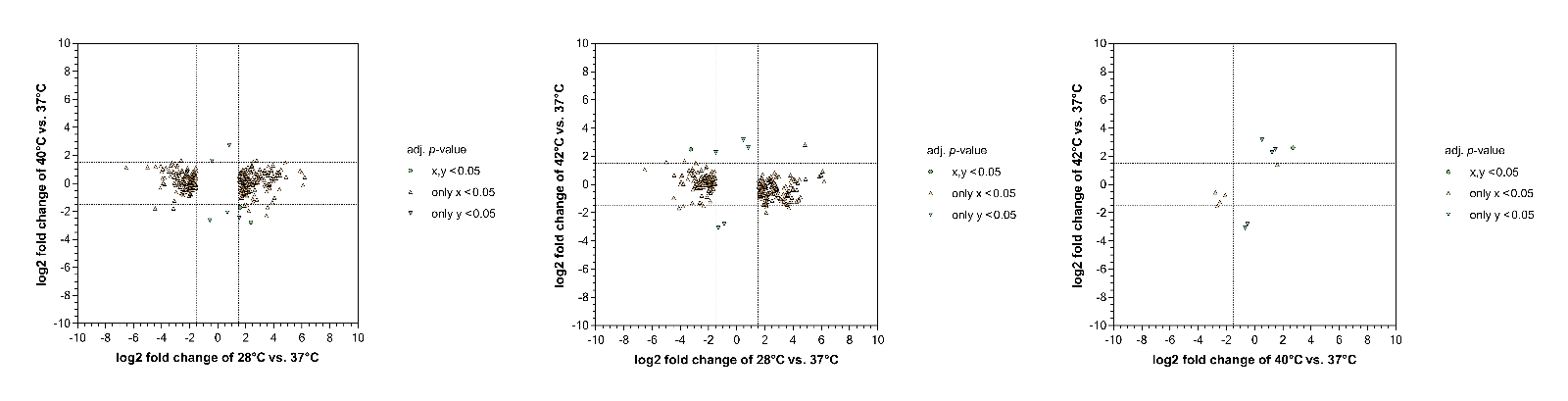

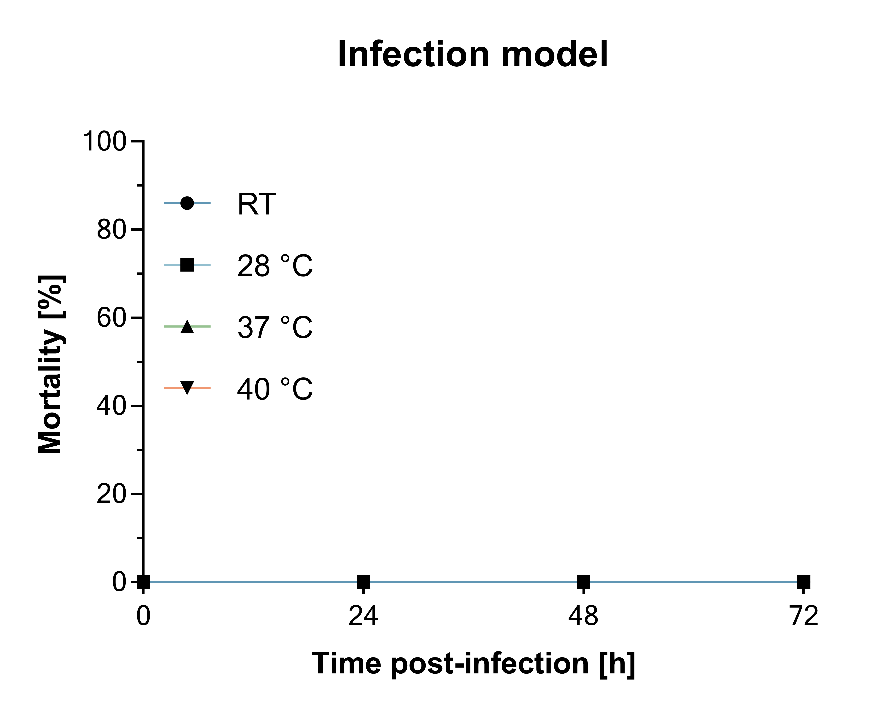
Supplementary Figure S3: The *Galleria mellonella* *in vivo* infection model with mock-infection (PBS) showed no temperature-dependent mortality rate at RT, 28 °C, 38 °C and 40 °C (Kaplan-Meier plot of mortality rates in the *G. mellonella* larvae [n = 30]). Results are expressed as mean percent mortality after injection of 10µL PBS.

Supplementary Figure S4: Correlation analysis between different temperatures to reveal the transcriptomic shift of 28 °C, 40 °C and 42 °C in comparison to 37 °C. Similar gene expression profiles in the upper-right and lower-left corners indicate transcriptomic changes independent of the selected media.


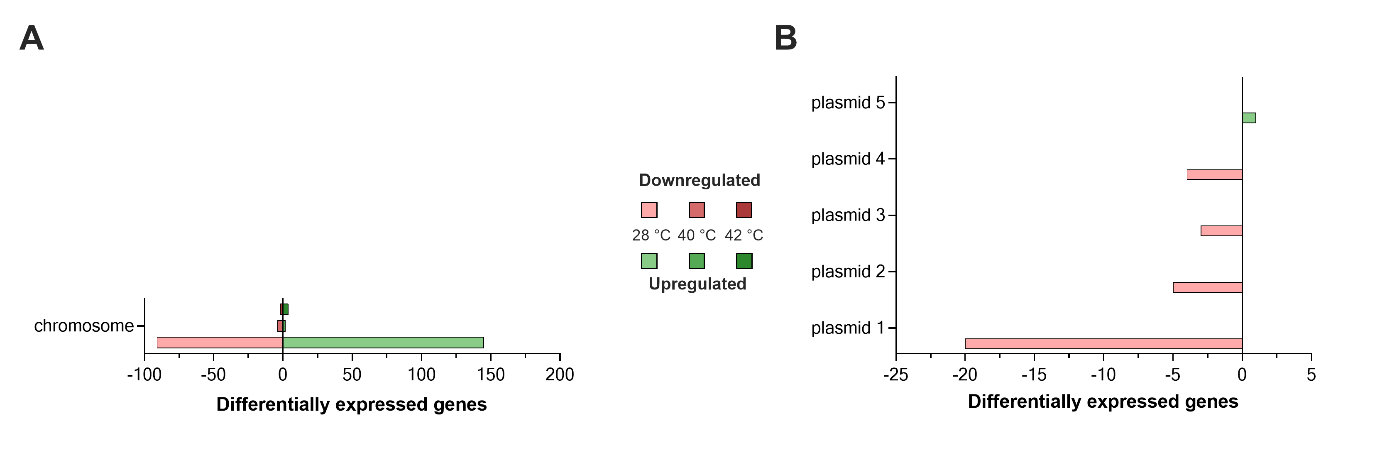


Supplementary Figure S5: RNA sequencing shows varying numbers of differentially expressed genes (e value < 0.05, |log2fold| change > 1.5) on the different encoded gene constructs at 28 °C, 40 °C, 42 °C compared to 28 °C in the chromosome (A) and in the different plasmids (B). Upregulated genes are colored in green, downregulated genes are colored in red.


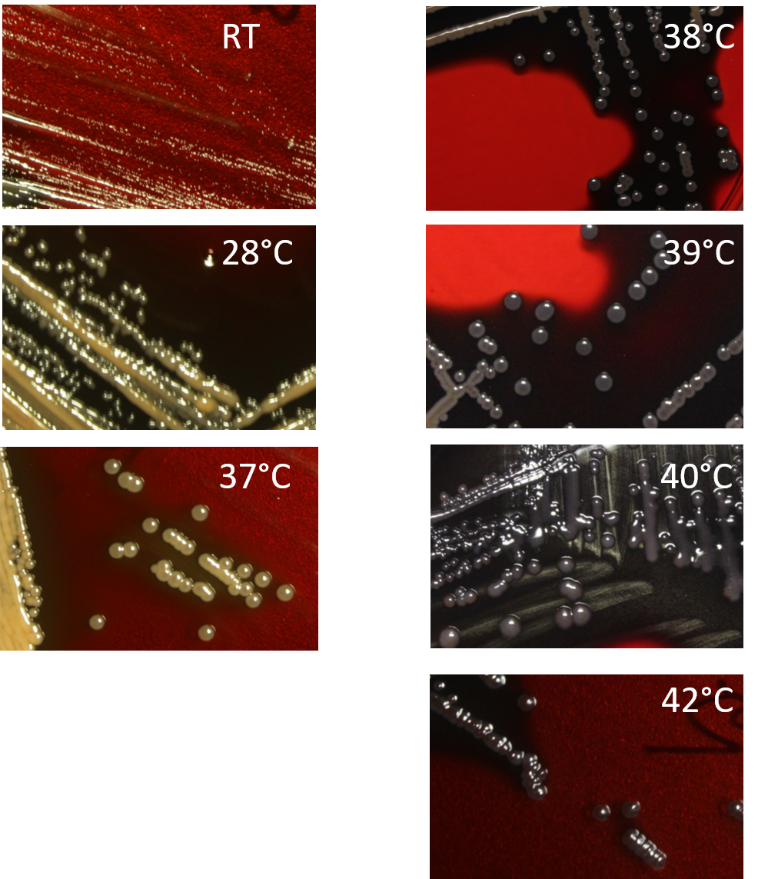
Supplementary Figure S6: Different temperatures affect mucoviscosity and overall virulence of the convergent *K. pneumoniae* ST307 strain PBIO1953. A staining of capsular polysaccharides revealed a temperature-dependent change from a “normal” mucoid phenotype (yellow-beige colonies) to a hypermucoid phenotype (black colonies) at 38 °C, 39 °C, 40 °C and 42 °C. The black color indicates extensive production of capsule polysaccharides and glycans.

*
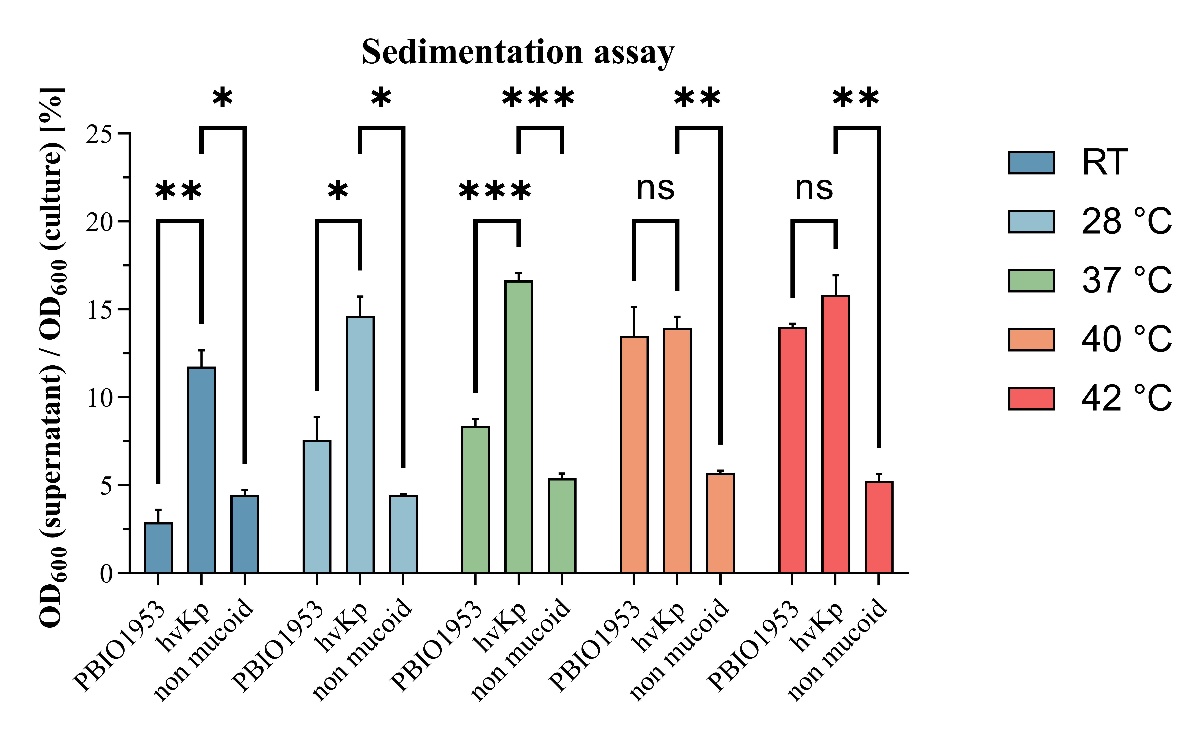
*

Supplementary Figure S7: **Different temperatures impact the viscosity of PBIO1953** in comparison to a hypervirulent *K. pneumoniae* (hvKp, highly mucoid, PBIO2030, ST420, KL20, O1/O2v1 (Eger et al., 2021)) and a non-mucoid control (PBIO1962, ST11, KL24, O1/O2v2 (Heiden et al., 2020)) (two-way ANOVA with Dunnett’s multiple comparison post hoc test; ns, not significant; P* <0.05; P** <0.01, P** < 0.001. RT, room temperature.

# Supplementary Tables

### Supplementary Table 1. Primer Sequences for plasmid copy number analyses of plasmid 1- 3, and chromosome.

| Name | Primer sequence | Amplicon length |
| --- | --- | --- |
| plasmid_1_forward | CGGAAGGAAGCCAGTACAGG | 141 bp |
| plasmid_1_reverse | TTGTCAACGAGGTCTGGACG |  |
| plasmid_2_forward | TTGCCGTTGTCTCACCTACC | 196 bp |
| plasmid_2_reverse | TGTACTGGCCACCTTCATCG |  |
| plasmid_3_forward | TGACATGGCAAAAGTTCAGGC | 83 bp |
| plasmid_3_reverse | CCTCAACTCGCCTCTTCTCC |  |
| chromo_forward | TCGCGATGAGTACAATCCGG | 82 bp |
| chromo_reverse | AAGGTCAACAGCAGGGTACG |  |

### Supplementary Table 2. qPCR reaction setup per well.

| Component | 20 µl reaction |
| --- | --- |
| Luna Universal qPCR Master Mix | 10 µl |
| Forward primer (10µM) | 0.5 µl |
| Reverse primer (10µM) | 0.5 µl |
| Template DNA | 50 ng |
| Nuclease free water | to 20µl |

### Supplementary Table 3. qPCR thermocycler protocol for the Biorad DFX Optus 96-well, qPCR Luna Mastermix.

| Cycle step | Temperature | Time | Cycles |
| --- | --- | --- | --- |
| Initial Denaturation | 95 °C | 60 s | 1 |
| Denaturation Extension | 95 °C 60 °C | 15 s 30 s (+plate read) | 45 |
| Meltingcurve | 60 °C -> 95 °C | Biorad CFX Optus standard | 1 |

# Supplementary methods

## Biofilm staining

The long-term colony assay, as described by (Schaufler et al., 2016), assesses bacterial biofilm formation. Span-ager plates were prepared and stained with congo red/coomassie-brilliant-blue solution (0.05 %/ 0.25 % 20 ml/l). Bacterial suspensions with an OD_600_ of 0.5 were then dropped onto the plates (5 µL per plate, maximum four samples). Plates were incubated at RT, 28°C, 37°C, 42°C for 5 days and visually evaluated.

# Supplementary literature

Eger, E., Heiden, S. E., Becker, K., Rau, A., Geisenhainer, K., Idelevich, E. A., & Schaufler, K. (2021). Hypervirulent Klebsiella pneumoniae Sequence Type 420 with a Chromosomally Inserted Virulence Plasmid. *International Journal of Molecular Sciences*, *22*(17). https://doi.org/10.3390/ijms22179196

Heiden, S. E., Hübner, N.-O., Bohnert, J. A., Heidecke, C.-D., Kramer, A., Balau, V., Gierer, W., Schaefer, S., Eckmanns, T., Gatermann, S., Eger, E., Guenther, S., Becker, K., & Schaufler, K. (2020). A Klebsiella pneumoniae ST307 outbreak clone from Germany demonstrates features of extensive drug resistance, hypermucoviscosity, and enhanced iron acquisition. *Genome Medicine*, *12*(1), 113. https://doi.org/10.1186/s13073-020-00814-6

Schaufler, K., Semmler, T., Pickard, D. J., de Toro, M., de La Cruz, F., Wieler, L. H., Ewers, C., & Guenther, S. (2016). Carriage of Extended-Spectrum Beta-Lactamase-Plasmids Does Not Reduce Fitness but Enhances Virulence in Some Strains of Pandemic E. coli Lineages. *Frontiers in Microbiology*, *7*, 336. https://doi.org/10.3389/fmicb.2016.00336
